# Perineal Wound Healing Following Abdominoperineal Resection of the Rectum

**DOI:** 10.7759/cureus.66318

**Published:** 2024-08-06

**Authors:** Muhammad Ali Khattak, Awais Nawaz Khan, Salman Jafferi, Yasir Iqbal, Habeeb Abdulrasheed, David McArthur

**Affiliations:** 1 Urology, Heartlands Hospital, University Hospitals Birmingham NHS Foundation Trust, Birmingham, GBR; 2 Orthopedics, Ghurki Trust Teaching Hospital, Lahore, PAK; 3 General Surgery, Heartlands Hospital, University Hospitals Birmingham NHS Foundation Trust, Birmingham, GBR; 4 Acute and General Medicine, Queen Elizabeth Hospital Birmingham, Birmingham, GBR

**Keywords:** complications, omentoplasty, risk factors, elape, perineal wound healing, abdomino-perineal resection, rectal cancer

## Abstract

Objective: This study aimed to investigate perineal wound healing rates following abdominoperineal resection (APR) or extralevator abdominoperineal excision (ELAPE) for rectal cancer, with a focus on identifying associated risk factors and outcomes.

Methodology: A retrospective analysis was conducted on patients undergoing APR or ELAPE for rectal cancer in a tertiary centre between 2013 and 2020. Data on demographics, comorbidities, surgical techniques, and perineal wound outcomes were collected and analyzed using Statistical Package for the Social Sciences (IBM SPSS Statistics for Windows, IBM Corp., Version 27.0, Armonk, NY). Statistical significance was set at p<0.05.

Results: A total of 87 patients were included, with a mean age of 64 years and the majority being male (66.7%). Neoadjuvant radiotherapy was administered in 87.4% of cases. Perineal wound complications were documented in 52 cases (59.8%), with major complications observed in 11 cases (12.6%). Healing within six months was achieved in 48 patients (55.2%), while 39 patients (44.8%) developed chronic perineal wounds. Logistic regression analysis revealed omentoplasty as a significant predictor of perineal wound healing rate showing a significant negative association (p=0.0289).

Conclusion: Perineal wound healing rates following APR or ELAPE varied. While most patients achieved complete healing, chronic perineal wounds presented challenges. Omentoplasty was associated with lower odds of healing, suggesting the need for further investigation into its role. These findings underscore the importance of patient counselling and multidisciplinary management strategies to optimize outcomes in rectal cancer surgery.

## Introduction

Rectal cancers are tumours found within 15 cm from the anal verge and it accounts for almost 30% of all colorectal malignancies [1]. Surgical intervention remains the cornerstone of curative treatment with the addition of neoadjuvant chemo and radiotherapy given in selected cases [2].

Surgery for lower rectal cancer involves a variety of techniques, each tailored to the specific characteristics of the tumour, patient factors and the goal of achieving optimal oncological and functional outcomes. Different surgical interventions used for low rectal cancers include low anterior resection, abdominoperineal resection (APE), extralevator abdominoperineal resection (ELAPE), intersphincteric resection, transanal total mesorectal excision and pelvic exenteration. ELAPE is recommended for advanced low rectal cancers [3,4].

ELAPE was introduced as an alternative to conventional APE in the treatment of low rectal cancers aiming to decrease the incidence of positive resection margins and intraoperative tumour perforations. One of the drawbacks is that with ELAPE there is more extensive resection which leaves a greater perineal defect increasing the risk of perineal wound complications, pain and hernia as compared to APE [5]. The incidence of perineal wound complications following APE and ELAPE is a topic of significant interest, and understanding the impact of this morbidity on patients is crucial for optimizing outcomes. Perineal wound complications can encompass a range of issues, including infections, dehiscence and delayed wound healing.

Studies have reported varying rates of perineal wound complications following both APE and ELAPE. In one of the observational studies, Asplund et al. in 2015 found that the incidence of perineal wound complications was 25% in all patients and was found more frequently in ELAPE than after conventional APE (32% vs 11%, p<0.001) [6].

Similarly in another Danish nationwide retrospective study done by Colov et al. in 2016, it was found that perineal wound complications were more frequent after ELAPE (44%) as compared to conventional APE (25%) [5]. One retrospective study done by Dsouza et al. in 2019 showed that perineal wound complications were seen in 63% of patients after conventional APE compared to 64.9% of the patients after ELAPE [7].

Several risk factors contribute to the development of perineal wound complications, including patient-related factors such as co-morbidities of diabetes mellitus, hypertension, steroid use, obesity, and surgical factors such as surgical technique, use of omentoplasty and biological mesh, and use of neoadjuvant therapy [8]. These perineal wound complications can have a significant impact on patients’ post-operative recovery and overall quality of life. Prolonged healing times, the need for additional interventions, and increased pain levels contribute to a challenging recovery period. As a result, patients experience psychological distress, which can potentially affect their emotional well-being. Sometimes wounds that evolve into sinuses need management, creating a continuous stressor for patients [9].

Management of these perineal wounds increases the economic burden on healthcare. It prolongs hospital stay contributing to escalated costs. Frequent dressings required to address these wounds further compound the expenses. Certain cases may involve more complex interventions such as debridement or surgical closure using flaps and grafts, further increasing the economic burden [10].

Achieving optimal healing for perineal wounds following APR or ELAPE poses considerable challenges. Preoperative optimization of the patient’s health, meticulous surgical technique, and post-operative wound care protocols can contribute to reducing the incidence of perineal wound complications. A multidisciplinary approach involving colorectal surgeons, wound care specialists, and other healthcare professionals can play a vital role in comprehensive management. Close monitoring of patients postoperatively can allow for early detection and interventions in cases of perineal wound complications. The aim of this study was to comprehensively examine the rates of perineal wound complications within our centre and to identify and understand the various factors involved in delayed wound healing, recognizing modifiable risk factors and implementing strategies to minimize them.

## Materials and methods

We performed a retrospective study of perineal wound healing rates in our tertiary centre, Heartlands Hospital, Birmingham. We included patients aged more than 18 undergoing APR or ELAPE for cancer. Patients who had surgery for a benign diagnosis, underwent intersphincteric perineal dissection, or died within 30 days were excluded. Additionally, patients who had flap reconstruction of the perineum were excluded, as data from this population has been previously published from our centre.

To identify a suitable cohort, we generated an electronic list of all operations performed by all consultants at our centre between the dates 2013 and 2020. Within this list, we searched for the codes APR, and ELAPE and for free text specifying APR or ELAPE. A total of 140 cases were retrieved, of which 53 were excluded. Among the excluded cases, 23 patients had surgery for causes other than cancer, 26 had flap reconstruction, and four had incomplete records. Operation notes were screened to ensure that the patients met the inclusion criteria.

This was a retrospective study that did not involve any changes to patient care so was registered as an audit and approval was taken from the Clinical Audit Registration and Management System (CARMS). We collected data on demographics, risk factors, surgical techniques, and perineal wound outcomes. All the data were entered and analyzed using Statistical Package for the Social Sciences (IBM SPSS Statistics for Windows, IBM Corp., Version 27.0, Armonk, NY) taking a p-value less than 0.05 as significant.

Descriptive statistics were used to summarize the baseline characteristics of the study population. Continuous variables, such as age and BMI, were reported as mean and standard deviation (SD). Categorical variables, such as gender, smoking status, steroid use, diabetes, neoadjuvant radiotherapy (NART), type of operation, surgical approach, omentoplasty, local antibiotics, and mesh use, were reported as counts and percentages. For analysis of outcome data, continuous variables such as duration of healing, were reported as median. Categorical variables, such as seven-day readmission, 90-day mortality, complications, and post-operative referrals, were reported as counts and percentages.

Independent t-test was used to compare normally distributed continuous variables between the two groups. The Mann-Whitney U test was used for non-normally distributed continuous variables. The chi-square test or Fisher's exact test was used to compare proportions between the two groups. A univariate logistic regression analysis was performed to assess the association between various predictor variables and the outcome of interest. The predictor variables included age, sex, BMI, smoking status, steroid use, type 2 diabetes mellitus (T2DM), NART, omentoplasty, use of perineal antibiotics, type of surgery (ELAPE), mesh use, and major complications excluding perineal complications. For each predictor variable, coefficient, odds ratio, 95% confidence interval, and p-value were reported.

## Results

The baseline characteristics of the study population (n=87) are summarized in Table 1. The mean age was 64 with an SD of ±14.5. The majority were male (n=58, 66.7%). Among the risk factors, the mean BMI was 28.7 kg/m^2^ with an SD of 4.9. Current smokers were 43.7% (38 individuals), six individuals reported steroid use (6.9%), and 15 individuals had a history of diabetes (17.2%). A significant majority underwent NART (87.4%, 76 individuals). Twenty-eight patients underwent APR (32.2%), 59 patients underwent ELAPE (67.8%); the surgical approach was open in 31% (27 cases), laparoscopic in 63.2% (55 cases), and in 5.7%, it was laparoscopic converted to open. Omentopalsty was done in 18.4% of cases, local antibiotics were used in 72.4% and biological mesh was used in 72.4% as shown in Table 1.

**Table 1:** Demographics and Baseline Characteristics Data APR: abdominoperineal resection; ELAPE: extralevator abdominoperineal excision

Category	Value	Percentage/Details
Total	87	
Demographics		
Age	64	±14.5
Male	58	66.7%
Risk Factors		
BMI	28.7	±4.9
Smokers	38	43.7%
Steroid use	6	6.9%
Diabetes	15	17.2%
Neoadjuvant radiotherapy	76	87.4%
Surgical Operation		
APR	28	32.2%
ELAPE	59	67.8%
Surgical Approach		
Open	27	31%
Lap	55	63.2%
Converted	5	5.7%
Other Factors		
Omentoplasty	16	18.4%
Local antibiotics	63	72.4%
Mesh use	63	72.4%

Among 87 patients, in 48 (55%) perineal wounds healed in less than six months but in 39 (45%) patients wound did not heal and became a chronic perineal wound. The seven-day readmission rate was 13.8% among all patients but 10.4% in those whose wounds healed within six months and 17.9% in those who had chronic perineal wounds. Overall, the 90-day mortality rate was 2.3% in all patients. Overall, 52 cases of complications were documented, constituting 59.8% of all patients. Among those with successful healing in less than six months, 23 patients (47.9) experienced complications, while 29 patients (74.4%) with chronic perineal wounds faced complications. Major complications were observed in 11 cases, representing 12.6% of the total patient population. In the subgroup of patients healed within six months, only two cases (4.2%) encountered major complications, whereas in the chronic perineal wound group, nine cases (23.1%) had major complications. Further classification of complications using the Clavien-Dindo (CD) grading system revealed varying degrees of severity. Notably, CD 2 complications were the most prevalent, with 28 cases (32.2%) in the overall patient population. In the subgroup of patients healed within six months, 12 cases (25%) fell into this category, while in the chronic perineal wound group, 16 cases (41%) were documented, as seen in Table 2.

**Table 2:** Outcome Data

Parameters		All patients	Healed <6 months	Chronic perineal wounds
Total		87	48	39
General				
Seven-day readmission		12 (13.8%)	5 (10.4%)	7 (17.9%)
Ninety-day mortality		2 (2.3%)	1 (2.1%)	1 (2.6%)
Complications				
All complications		52 (59.8%)	23 (47.7%)	29 (74.4%)
Major complications		11 (12.6%)	2 (4.2%)	9 (23.1%)
Clavien-Dindo 1		13 (14.9%)	9 (18.8%)	4 (10.3%)
Clavien-Dindo 2		28 (32.2%)	12 (25.0%)	16 (41.0%)
Clavien-Dindo 3A		3 (3.4%)	1 (2.1%)	2 (5.1%)
Clavien-Dindo 3B		7 (8.0%)	1 (2.1%)	6 15.4%)
Clavien-Dindo 4A		1 (1.1%)	0 (0.0%)	1 (2.6%)
Clavien-Dindo 4B		0 (0.0%)	0 (0.0%)	0 (0.0%)
Persistent perineal sinus		11 (12.6%)	0 (0.0%)	11 (28.2%)
Re-operation for perineal complication		4 (4.6%)	0 (0.0%)	4 (10.3%)
Perineal hernia		4 (4.6%)	2 (4.2%)	2 (5.1%)
Perineal Wound Healing				
Healing by the first appointment		39 (44.8%)	39 (81.3%)	0 (0.0%)
Reached complete healing		71 (81.6%)	48 (100.0%)	23 (59.0%)
Median duration of healing (days)		75 (167.75)	56.5 (45.75)	414 (211.25)
Post-operative Referrals				
Plastic surgery		4 (4.6%)	2 (4.2%)	2 (5.1%)
Dressings clinic		19 (21.8%)	7 (14.6%)	12 (30.8%)
Tissue viability nursing		4 (4.6%)	1 (2.1%)	3 (7.7%)
Topical negative pressure therapy		4 (4.6%)	0 (0.0%)	4 (10.3%)

Specific complications such as persistent perineal sinus, re-operation for perineal complications and perineal hernia were also examined. Persistent perineal sinus occurred in 11 cases (12.6%), exclusively in the chronic perineal wound group. Reoperation for perineal complication was seen in four cases in the chronic perineal wound subgroup. The perineal hernia was observed in a total of four cases of which two were in the subgroup whose wounds healed within six months and two cases in the subgroup of chronic perineal wounds as seen in Table 2.

In terms of perineal wound healing, 39 patients (44.8%) achieved healing by the first appointment, with this success notably higher in the subgroup of patients healed within six months, where 39 individuals (81.3%) experienced complete healing. Strikingly, none of the patients with chronic perineal wounds achieved complete healing by the first appointment as seen in Table 2.

Overall, 71 patients (81.6%) reached complete healing, with 48 patients (100%) achieving this outcome in the group healed within six months and 23 patients (59%) in the chronic perineal wound group. The duration of healing, measured in days, varied across the patient population, with a median of 75 days. The average days of healing in the subgroup within six months was 56.5 days and the average duration of healing in the chronic perineal wound group was 414 days.

Concerning post-operative referrals to plastic surgery, consultations were sought for four cases (4.6%). Of these, two cases were in the group that healed within six months, and two cases were in the chronic perineal wound group. Dressings clinic referrals were more frequent, accounting for 19 cases (21.8%), with seven cases (14.6%) in the subgroup healed within six months and 12 cases (30.8%) in the chronic perineal wound group. Tissue viability nursing referrals were made in four cases (4.6%), predominantly in the group that healed within six months. Topical negative pressure dressing was utilized in four cases (4.6%) exclusively in the chronic perineal wound group.

Age, sex, BMI, smoking, steroids, T2DM, radiotherapy, omentoplasty, perineal antibiotic use, type of procedure, mesh usage, and major complications excluding perioperative complications showed no significant association with perineal wound healing rates. Notably, omentoplasty exhibits a significant negative association (odds ratio: 0.22, 95% CI: 0.05-0.86; p=0.03), indicating a lower odd of the outcome. These findings provide valuable insights into the potential factors influencing perineal wound healing rates post-ELAPE, emphasizing the potential impact of omentoplasty (Table 3).

**Table 3:** Relationship Between Risk Factors and Wound Healing T2DM: type 2 diabetes mellitus; NART: neoadjuvant radiotherapy; ELAPE: extralevator abdominoperineal resection

Variable	Odds ratio (OR)	Confidence interval (95% CI)	P-value
Age	0.98	0.94-1.02	0.45
Sex	0.51	0.20-1.34	0.17
BMI	0.96	0.88-1.06	0.44
Smoking	0.84	0.34-2.06	0.70
Steroids	2.43	0.42-14.18	0.32
T2DM	2.44	0.44-1.59	0.21
NART	0.67	0.17-2.71	0.58
Omentoplasty	0.22	0.05-0.86	0.03
Local antibiotics	0.95	0.06-2.60	0.93
ELAPE	0.40	0.15-1.06	0.07
Mesh	1.10	0.40-1.12	0.86
Major complications	0.17	0.02-0.38	0.11

## Discussion

In our study, the mean age was 64 ± 14.5 and the majority of the patients were male 58 (66.7%). The mean BMI was 28.7 ± 4.9, smoking was observed in 38 (43.7%) individuals and steroid use was seen in six people (6.9%). Fifteen (17.2%) cases had diabetes and 76 (87.4) had NART prior to surgery. Twenty-seven (31%) had open surgeries and 55 (63.2%) had laparoscopic surgeries; only five cases of laparoscopic approach were converted into open. Omentoplasty was done in 16 (18.4%) cases and local antibiotics and mesh were used in 63 (72.4%) cases each. There was no significant association found between age, sex, BMI, smoking status, and perineal wound healing and the results were not statistically significant as well.

There was no significant association found between perineal wound healing and the use of steroids, local use of antibiotics, diabetes, and NART. In our study, complete healing was achieved in 71 cases (81.6%) and the average healing time was 75 days. In a similar retrospective study done by Althumairi et al. on 175 patients delayed wound healing was observed in 44 (25%) of patients with mean time to heal around 6.3 weeks [11]. In another study done by Chang et al. delayed wound healing was observed in 86 (37.6%) cases and the median perineal wound healing time was 63.5 days [12]. In our study univariate logistic regression showed that the use of omentoplasty was associated with a lower chance of healing by the first appointment (OR 0.22, 95% CI: 0.05-0.85, p=0.02); however, in another retrospective study done by Nagata et al. in 2019, it was observed that omentoplasty decreases the risk of perineal wound infection and dehiscence (78.6% vs 46.4%, p<0.001) [13]. Similarly in another systematic review and meta-analysis done by Blok et al. on omentoplasty for the management of abdominoperineal defects in patients treated for cancer, no association was found between omentoplasty and perineal wound healing rate; there was an increased risk of perineal hernia among patients who underwent omentoplasty [14]. In a systematic review by Kim et al., findings revealed no significant differences in wound healing rates at one and three months post-operatively between the omental transposition and control groups. Rates of perineal wound infection and chronic complications, including chronic sinus formation, perineal wound dehiscence, and fistula, also showed no significant differences [15]. In a meta-analysis conducted by Lu et al., it was found that omentoplasty significantly reduces the morbidity associated with perineal wound complications [16].

Perineal wound healing success was notably higher (81.3%) in the subgroup healed within six months on their first follow-up appointment in the clinic, while none of the chronic wound cases achieved complete healing at the first appointment. Referrals for plastic surgery, dressings clinic, tissue viability nursing, and specialized interventions like topical negative pressure therapy were made based on post-operative considerations. These results of perineal wound healing can be used to inform patients during the consent process for surgeries such as ELAPE and APR. They provide patients with a clearer understanding of the broader spectrum of morbidity associated with these procedures.

This was a single-centre study with a smaller sample size, and the average length of stay was not observed but could be crucial in understanding the overall patient experience. To clarify the role of omentoplasty in perineal wound healing, further prospective studies can be done.

## Conclusions

The management of low rectal cancers presents a complex clinical challenge, particularly with the choice of surgical technique impacting patient outcomes significantly. ELAPE offers an advantage in reducing positive resection margins and intraoperative tumour perforations but is associated with a higher risk of perineal wound complications compared to conventional APE. Our study analysis revealed no significant associations between common risk factors (age, sex, BMI, smoking, steroids, diabetes, and NART) and perineal wound healing outcomes, except for the negative impact of omentoplasty on healing rates. This finding aligns with some literature while contradicting others, indicating the need for further research to clarify omentoplasty's role in perineal wound management. The higher complication rates and the extended healing duration in the chronic wound group emphasize the necessity for enhanced post-operative care protocols and the potential benefits of a multidisciplinary approach involving colorectal surgeons, wound care specialists, and other healthcare professionals.

In conclusion, achieving optimal perineal wound healing after ELAPE or APE surgery needs proper pre-operative planning, precise surgical technique, and thorough post-op care. Our study shows the importance of ongoing research to find better ways to prevent and treat these wound complications. This will help improve patient recovery and reduce healthcare costs. Further studies with larger sample sizes and multi-centre collaboration are needed to confirm our findings and make management protocols for patients undergoing surgery for low rectal cancers.
